# Quantitative proteomic characterization of cellular pathways associated with altered insulin sensitivity in skeletal muscle following high-fat diet feeding and exercise training

**DOI:** 10.1038/s41598-018-28540-5

**Published:** 2018-07-16

**Authors:** Maximilian Kleinert, Benjamin L. Parker, Thomas E. Jensen, Steffen H. Raun, Phung Pham, Xiuqing Han, David E. James, Erik A. Richter, Lykke Sylow

**Affiliations:** 1 Department of Nutrition, Exercise and Sports, Faculty of Science, University of Copenhagen, Copenhagen, Denmark; 2 Institute for Diabetes and Obesity, Helmholtz Diabetes Center at Helmholtz Zentrum München, German Research Center for Environmental Health (GmbH), 85764 Neuherberg, Germany; 3 Division of Metabolic Diseases, Department of Medicine, Technische Universität München, 80333 Munich, Germany; 4 German Center for Diabetes Research (DZD), 85764 Neuherberg, Germany; 5 The University of Sydney, Charles Perkins Centre, School of Life and Environmental Sciences, Sydney, Australia

## Abstract

Regular exercise elicits advantageous metabolic adaptations in skeletal muscle, such as improved insulin sensitivity. However, the underpinning molecular mechanisms and the effect of diet on muscle exercise training benefits are unclear. We therefore characterized the skeletal muscle proteome following exercise training (ET) in mice fed chow or high-fat diet (HFD). ET increased exercise performance, lowered body-weight, decreased fat mass and improved muscle insulin action in chow- and HFD-fed mice. At the molecular level, ET regulated 170 muscle proteins in chow-fed mice, but only 29 proteins in HFD-fed mice. HFD *per se* altered 56 proteins, most of which were regulated in a similar direction by ET. To identify proteins that might have particular health-related bearing on skeletal muscle metabolism, we filtered for differentially regulated proteins in response to ET and HFD. This yielded 15 proteins, including the major urinary protein 1 (MUP1), which was the protein most decreased after HFD, but increased with ET. The ET-induced *Mup1* expression was absent in mouse muscle lacking functional AMPK. MUP1 also potentiated insulin-stimulated GLUT4 translocation in cultured muscle cells. Collectively, we provide a resource of ET-regulated proteins in insulin-sensitive and insulin-resistant skeletal muscle. The identification of MUP1 as a diet-, ET- and AMPK-regulated skeletal muscle protein that improves insulin sensitivity in muscle cells demonstrates the usefulness of these data.

## Introduction

Regular exercise has beneficial effects on most organs of the body^1^ and current public health organizations promote exercise as a cornerstone in prevention, management, and treatment of numerous chronic conditions, including hypertension, obesity, coronary heart disease, type 2 diabetes mellitus, and age-related muscle wasting^2^. The mechanisms underpinning these benefits are complex. Notably, adaptations in exercised skeletal muscle play a key role in locomotion, exercise performance, metabolic responses and regulation of whole body metabolic homeostasis^3^. Skeletal muscle comprises ∼40% of total body mass and accounts for ∼30% of the resting metabolic rate in adult humans^4^. Importantly, it is also the predominant (∼80%) site of glucose disposal under insulin-stimulated conditions^5^. Accordingly, it is not surprising that insulin-resistant muscle represents a major challenge to maintaining healthy glycaemia and thus is a risk factor for developing diabetes. Exercise improves insulin sensitivity of skeletal muscle, which translates to whole-body glycemic benefits^6,7^. This beneficial effect of exercise is recognized in the treatment and prevention of type 2 diabetes, since exercise is often more successful than pharmacological interventions^8^.

Some key exercise signaling proteins have been identified. For example, the 5′ AMP-activated protein kinase (AMPK) has been shown to be an essential signaling nexus for exercise-induced increase in insulin sensitivity^9,10^, while the peroxisome proliferator-activated receptor gamma coactivator 1-alpha (PGC-1α) is important for mitochondrial biogenesis^11,12^. Notwithstanding these important discoveries, recent studies have demonstrated that the signaling response to an acute exercise bout is far more complex than previously assumed^13–16^. Whether changes in the protein landscape following chronic exercise are equally dramatic is relatively unknown. Identifying the molecular mechanisms underlying improved insulin sensitivity following exercise training could provide novel therapeutic targets to treat insulin-resistant and type-2 diabetics.

In rodents, a high-fat diet can lead to obesity and insulin resistance and thus represents a model for the human metabolic syndrome^17^, an established risk factor for development of type 2 diabetes. Therefore, amelioration of insulin resistance by physical activity might be particularly relevant in conditions of obesity and insulin resistance. Furthermore, the current obesity crisis can in part be attributed to a pervasive availability of palatable, calorie-dense foods and the composition of the diet consumed during training influences both enzymatic adaptations and improvement in exercise performance^18,19^. This makes it relevant to understand how ET interacts with diet on the molecular level. Accordingly, we profiled adaptations in the skeletal muscle proteome of mice after 20 weeks of ET and exposure to either chow or HFD. Both ET and HFD increase the lipid oxidative capacity in skeletal muscle^20–22^. However, adaptations in lipid oxidative capacity are unlikely candidates for the specific beneficial effects of exercise, since only ET-, not HFD-induced lipid adaptations promote health benefits. A side-by-side comparison of the ET- and the HFD-controlled proteome can identify proteins that are differentially regulated by HFD and ET, thereby generating a unique list of candidate proteins that could have particular health-related bearing on skeletal muscle metabolism.

## Results

**Figure 1:**
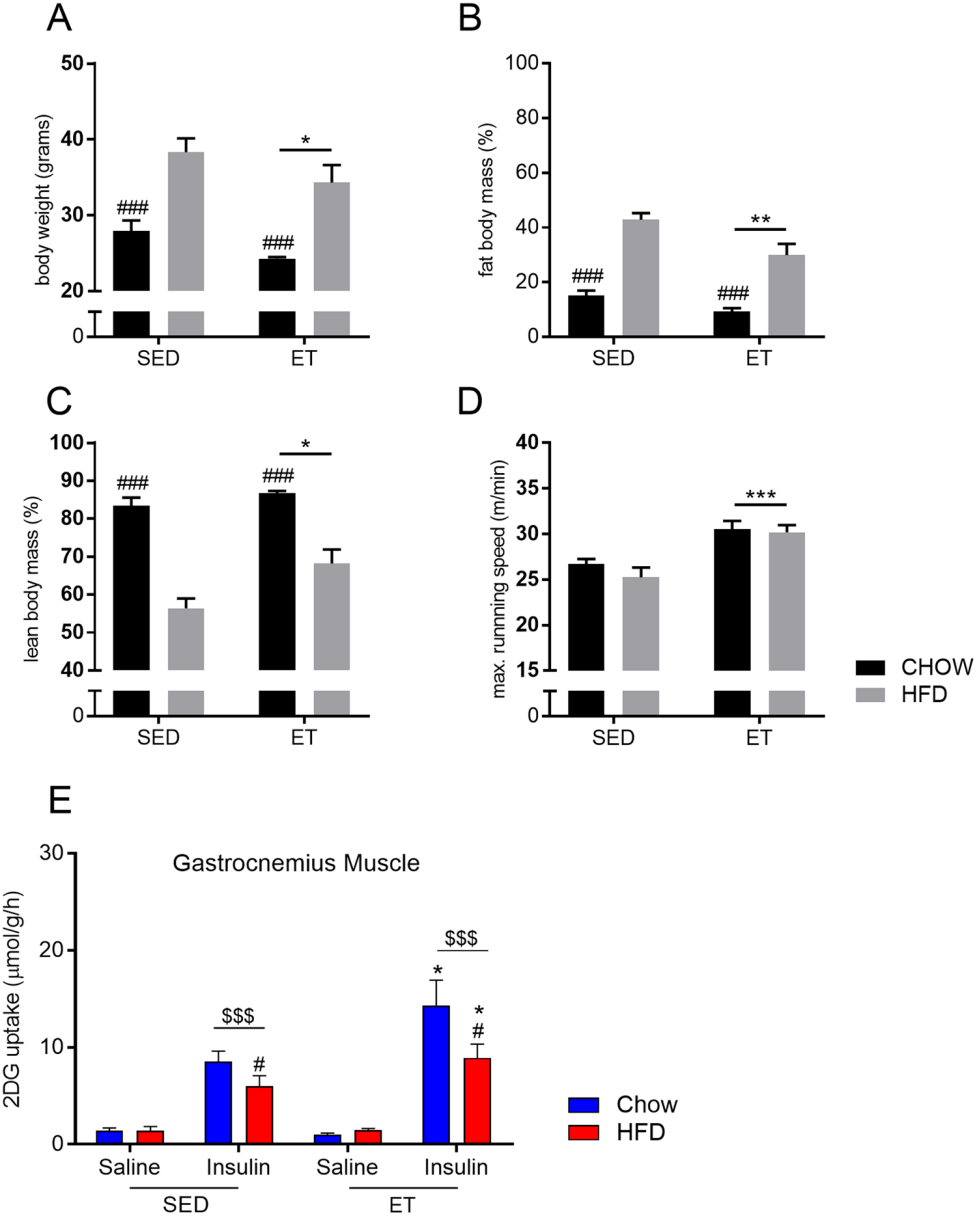
Exercise training (ET) improves running performance, body composition and insulin sensitivity. Mice with (ET) or without (sedentary (SED)) access to running wheels were fed either a chow (CHOW) or a high-fat diet (HFD) for 20 weeks and body weight (**A**), body composition (**B**,**C**), and maximal running capacity (**D**) were determined. *In vivo* saline- and insulin-stimulated glucose uptake into gastrocnemius muscle was assessed (**E**) following the 20 week long experimental period. Data are means +/− SEM (n = 4–5 for A-D and n = 5–8 for E). *p < 0.05, **p < 0.01 and ***p < 0.001 are main effects of ET (vs. SED). ^###^p < 0.001, ^#^p < 0.05 are main effects of diet (CHOW vs HFD). ^$$$^p < 0.001 is a main effect for insulin (Insulin vs Saline).

Sedentary (SED) and ET mice were fed either chow or HFD for 20 weeks. SED mice fed a HFD were ∼25% heavier compared to SED chow-fed mice (Fig. 1A). Both ET groups ran the same distance per day (∼6 km/day) (data not shown). ET lowered body weight in both diet groups (−7%) (Fig. 1A). Fat mass accounted for ∼40% of body mass in HFD-fed mice and for 12% in chow-fed mice (Fig. 1B). ET reduced fat mass and increased lean body mass in both diet groups (Fig. 1B,C). Maximal running speed increased with ET, independent of diet (+17%) (Fig. 1D). HFD decreased insulin action in muscle and importantly, ET increased *in vivo* insulin-stimulated glucose uptake into skeletal muscle in both diet groups (Fig. 1E), demonstrating a clear benefit of ET on muscle insulin action irrespective of diet.

**Figure 2:**
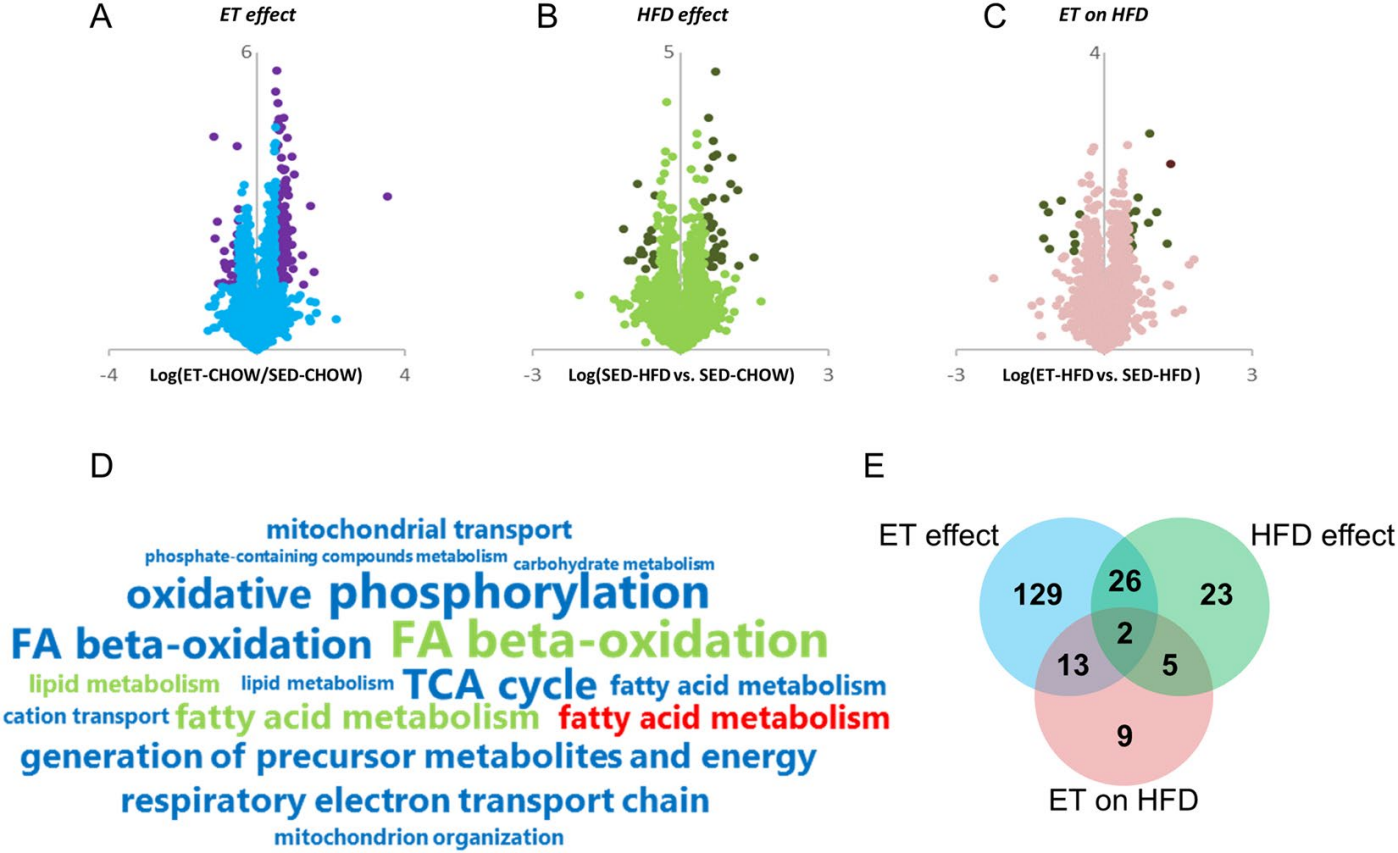
Exercise training (ET)- and high-fat diet (HFD)-regulated skeletal muscle proteins. Volcano plots for the ET effect (ET CHOW/ SED CHOW), the HFD effect (SED HFD/ SED CHOW) and the ET during HFD effect (ET HFD/ SED HFD) are shown (**A–C**) (darker circles within each plot are significantly altered (q < 0.05 and fold-change +/−50%). Enriched PANTHER GO-Slim Biological Process terms (**D**) based on the regulated proteins (blue, green and red font denote ET, HFD, and ET on HFD effects, respectively; the larger the font size the greater the log10 enrichment for the specified pathway). Venn diagram (**E**) of the altered proteins. SED = sedentary.

**Figure 3:**
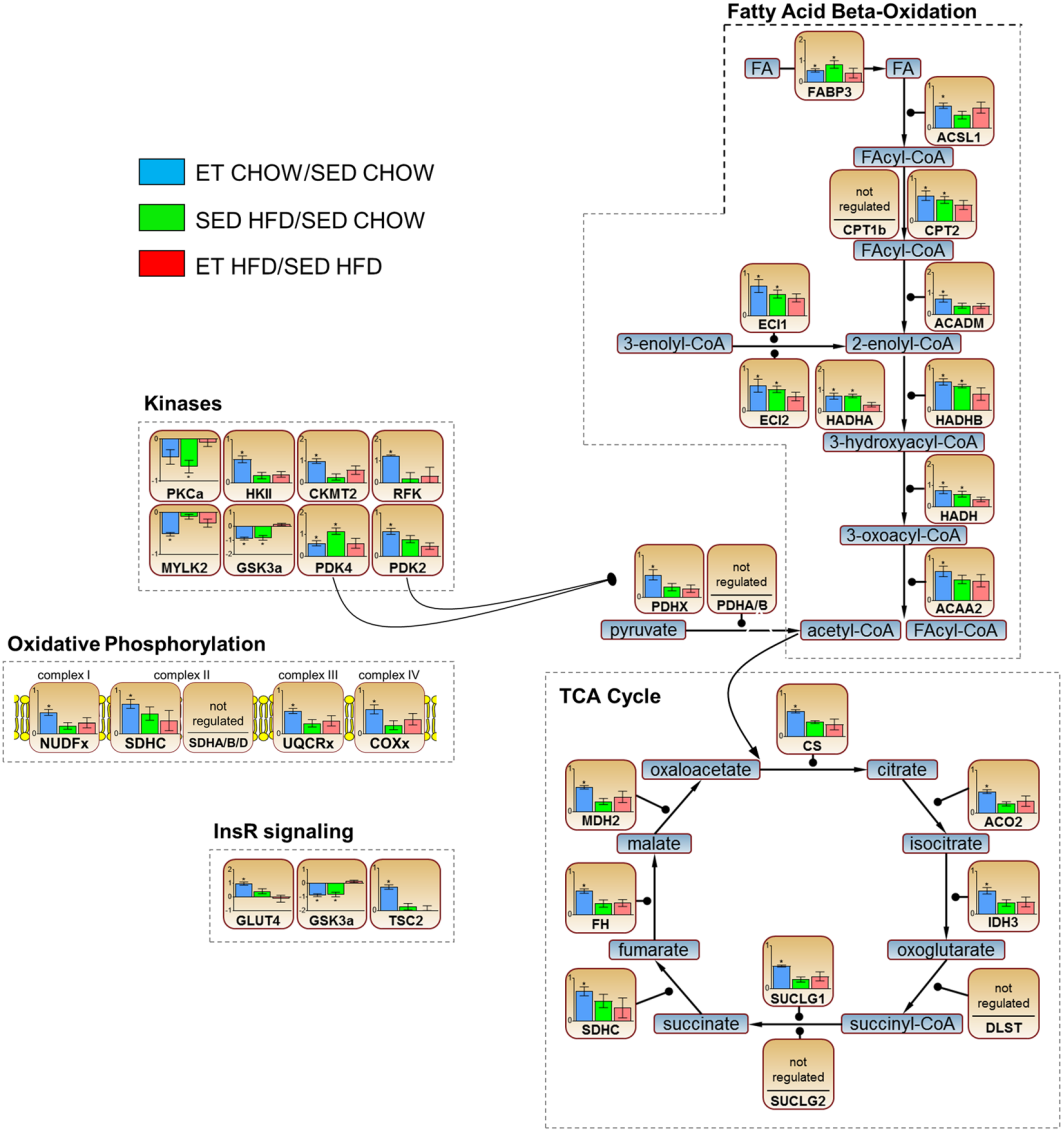
Metabolic pathways and protein groups regulated by exercise training (ET) and high-fat diet (HFD). Different proteins belonging to outlined cellular pathways or protein groups. Data are log2 fold changes of the indicated ratios +/− SEM (E-I). *q < 0.05 for the ratio. SED = sedentary; InsR = insulin receptor.

Proteomic analysis of gastrocnemius muscles identified 2,884 proteins at a 1% peptide and protein false discovery rate (FDR) with 2,391 proteins quantified in at least two biological replicates (Supplemental Table 1). ET in chow-fed mice altered 170 skeletal muscle proteins compared to SED chow-fed animals (q < 0.05 and fold-change +/−50%). Of those, 146 were up- and 24 down-regulated (Fig. 2A). Among these regulated proteins were known ET-responsive proteins, like GLUT4 (+90%) and hexokinase II (HKII; +50%) (Fig. 3). Significantly enriched gene ontology (GO) biological terms in ET chow-fed mice were dominated by biological functions related to fatty acid metabolism, oxidative phosphorylation, and mitochondrial adaptations (Fig. 2D). HFD altered the abundance of 56 proteins (39 up- and 17 down-regulated) compared to SED chow-fed mice (q < 0.05 and fold-change +/−50%) (Fig. 2B). Significantly enriched GO biological terms for the HFD-regulated proteins implicated changes in lipid metabolism pathways (Fig. 2D). ET during HFD feeding (ET on HFD) altered only 29 proteins (20 up- and 9 down-regulated) compared to SED HFD-fed controls (Fig. 2C) and the only significantly enriched GO biological term for ET on HFD was fatty acid metabolism (Fig. 2D).

**Figure 4:**
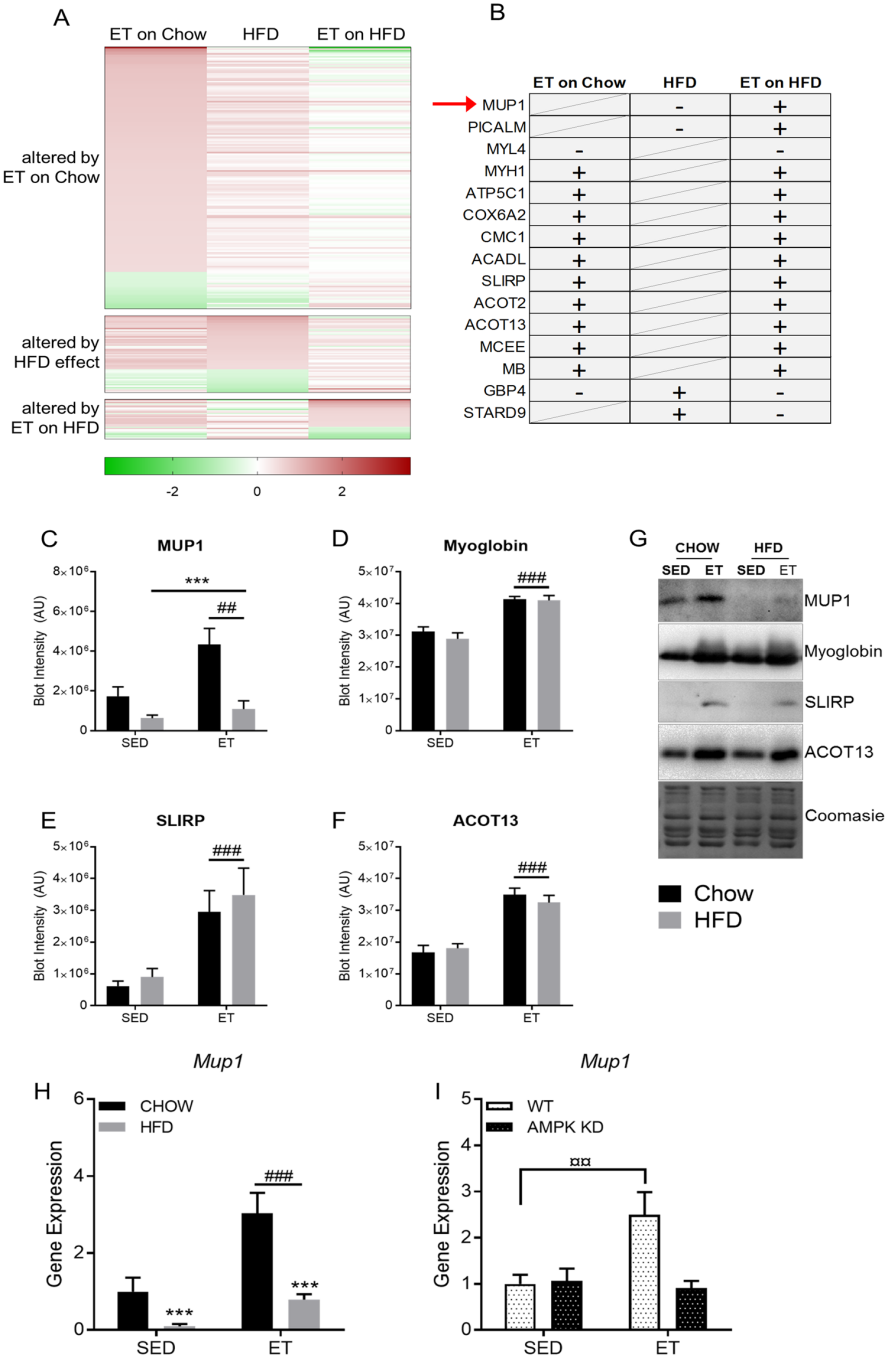
MUP1 is regulated by high-fat diet (HFD) and exercise training (ET). Heatmaps (**A**) of proteins altered (q < 0.05 and fold-change +/−50%) by ET on Chow (top), HFD (middle), or ET on HFD (bottom heatmap) with corresponding proteins from the other groups. Proteins depicted in (**B**) were either altered in the same direction by ET and ET on HFD, or they were oppositely altered by HFD and ET on Chow or ET on HFD (‘+’ denotes increased, ‘−’ decreased abundance; a diagonal strikethrough means that the protein was not significantly regulated). Quantification and representative images of western blot performed for the indicated proteins (**C–G**, n = 5–10). The effects of ET and HFD on *Mup1* gene expression in muscle (H, n = 7–10), as well as the effect of ET effect on *Mup1* gene expression in AMPK KD mice (I, n = 8). Data are means +/− SEM. ^***^p < 0.001 main effect of diet (HFD vs Chow). ^###^p < 0.001 and ^##^p < 0.01 main effects for activity (ET vs sedentary (SED)). ¤¤p < 0.01 difference between indicated groups.

There was a considerable overlap between the proteins regulated by HFD and by ET, as *ET* altered 26 of the 56 proteins altered by HFD in the same direction (Fig. 2E, Fig. 4A). For example, PDK4, which promotes FA oxidation by inhibiting the PDH-mediated conversion of glycolysis-derived pyruvate into acetyl-CoA^23^, was increased with both HFD and ET (Fig. 3). PDK4 is often considered a key regulator of metabolic flexibility allowing the muscle to quickly adjust to different fuel sources, like fatty acids (FAs) and glucose (reviewed in^24^). FABP3, which is involved in intracellular fatty acid transport^25^, was also increased with both HFD and ET (Fig. 3). These changes in PDK4 and FABP3 protein abundance are consistent with several studies showing that both exercise and HFD feeding increase fatty acid metabolic capacity^20,21^. This similarity is also evident in the GO terms, as the three biological pathways enriched in the HFD-regulated proteome (lipid metabolism, FA metabolism, and FA beta-oxidation) were enriched to a similar extent with ET (Fig. 2D).

These commonalities help explain why ET resulted in fewer regulated proteins during a HFD (compared to SED HFD mice). This makes the 29 proteins regulated by ET on HFD particularly interesting, because these are associated with improvements in insulin sensitivity based on enhanced insulin action in muscle (Fig. 1E) and their regulation by ET is likely independent of HFD-related processes. In addition, given the considerable overlap of HFD- and ET-regulated proteins, we surmised that the proteins underpinning the detrimental effects of HFD or, conversely, the beneficial effects of ET are the ones differentially regulated by HFD and ET. Such divergently regulated proteins would correlate with impaired and improved insulin sensitivity, making a causal relationship more likely, albeit not definite.

To this end, we sought to identify candidate proteins underpinning the exercise benefit (e.g. improved insulin sensitivity) by filtering for proteins that were either regulated by ET irrespective of diet or that were differentially regulated by HFD and ET (i.e. down with HFD but up with ET, or vice versa). This yielded 15 proteins (Fig. 4B). We tested proteins for which already validated antibodies were available and found that protein abundance of Myoglobin (MB), SLIRP and ACOT13 all increased with ET irrespective of diet (Fig. 4D–G) confirming the proteome data. The proteome data also indicated that the major urinary protein 1 (MUP1) was the most decreased protein on HFD, while ET blunted this HFD effect (Fig. 4B, Fig. S1A). This was confirmed by western blotting, which showed a marked decrease in MUP1 on HFD, while ET increased MUP1 protein abundance (Fig. 4C). The detection of MUP1 protein in skeletal muscle was intriguing, because MUP1 is reported to be a liver-specific protein that may play a role in regulating insulin sensitivity^26,27^. A possible explanation is that we had detected liver-secreted MUP1 taken up from the circulation by skeletal muscle. However, *Mup1* transcripts were detected in cultured mouse C2C12 muscle cells and in mouse quadriceps muscle (Fig. S1B), suggesting that *Mup1* is endogenously expressed in mouse skeletal muscle. Compared to the liver, skeletal muscle *Mup1* gene expression is substantially lower, but still robust and several magnitudes higher compared to levels of *Mup1* in the hypothalamus (Fig. S1B,C). In line with the alterations in protein abundance, skeletal muscle *Mup1* gene expression was 10-fold lower after 20 weeks of HFD, while ET had the opposite effect, increasing *Mup1 gene* expression 3-fold and 8-fold in chow-fed and HFD-fed mice respectively (Fig. 4H).

Having found that MUP1 is an ET- and HFD-regulated protein in skeletal muscle, we next investigated upstream regulation. A key regulator of energy homeostasis that is activated by exercise and required for many of the exercise-related health benefits is AMPK^28^. We therefore investigated whether the increased *Mup1* expression with ET requires functional AMPK by measuring *Mup1* gene expression in skeletal muscle from SED and ET wildtype (WT) or transgenic mice overexpressing an AMPKα2 kinase dead (AMPK KD) construct in striated muscle^29^. AMPK KD mice exhibit a reduced running capacity, but with ET both WT and AMPK KD mice can adapt to ET and improve their running capacity to similar extent^30^. In SED WT and SED AMPK KD mice, *Mup1* expression was similar (Fig. 4E). With ET, *Mup1* expression increased in WT (+250%), but not in AMPK KD mice (Fig. 4E), suggesting that AMPK activation is required for the ET-mediated increase in skeletal muscle *Mup1* gene expression.

**Figure 5:**
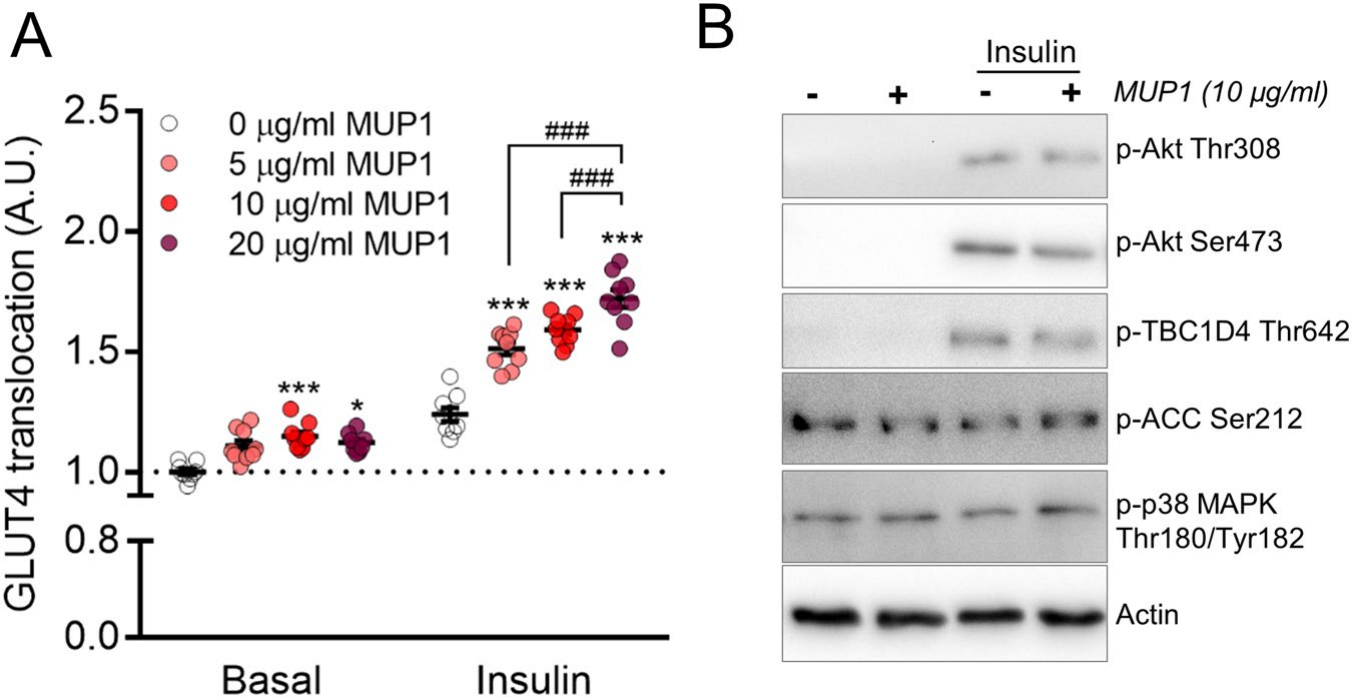
MUP1 augments insulin-stimulated GLUT4 translocation in cultured muscle cells. Insulin-stimulated (10 nM) GLUT4 translocation (**A**) and signaling (**B**) was assessed in L6-mycGLUT4 myoblasts in presence of the indicated concentrations of recombinant MUP1 protein. In A data are means +/− SEM. ***p < 0.001 and *p < 0.05 different from 0 µg/ml within Basal or Insulin. ^###^p < 0.001 differences between indicated groups. In B, representative results of three independent experiments are shown.

The induction by ET, possibly via AMPK, makes MUP1 an intriguing candidate that may play a role in the insulin sensitizing effects of exercise. In support of the notion, a previous study reported an improved glucose tolerance in obese diabetic mice following pharmacological treatment with recombinant MUP1^27^, but it is unknown whether this reflected improved insulin sensitivity at the level of the muscle. To understand whether MUP1 has a direct effect on muscle insulin sensitivity, we employed L6 muscle cells that stably overexpress a myc-tagged version of the insulin-responsive glucose transporter 4 (GLUT4). This allows for quantification of GLUT4 translocation from the inside of the cell to the membrane and this model has been widely used to investigate alterations in insulin sensitivity. When we pretreated these cells with recombinant MUP1 protein, basal GLUT4 translocation was increased (Fig. 5A). Furthermore, MUP1 potentiated submaximal insulin-mediated GLUT4 translocation in a dose-dependent manner, suggesting that MUP1 has direct insulin sensitizing properties in muscle cells (Fig. 5A). Akt signaling, AMPK activity (judged by p-ACC Ser212), and p38 phosphorylation were not altered by MUP1 (Fig. 5B), suggesting that these pathways do not mediate the effect of MUP1 on GLUT4 translocation.

## Discussion

In the present study, we provide a unique resource of proteins regulated by exercise training (ET), high-fat diet (HFD), and ET during HFD in mouse skeletal muscle. This side-by-side comparison allowed us to create a list of candidate proteins that may be particularly relevant for transducing the beneficial metabolic adaptions of exercise. The usefulness of this resource is demonstrated by the identification of MUP1 as a HFD- and ET-regulated skeletal muscle protein that improves insulin sensitivity in muscle cells.

In healthy insulin-sensitive individuals, 50–60% of carbohydrates in a meal are taken up by skeletal muscle^31^. With this large contribution to postprandial glucose disposal, insulin resistance of skeletal muscle^5^ represents a serious challenge to whole body glucose homeostasis. Consequently, maintaining muscle mass and metabolic function is essential for health. Exercise improves skeletal muscle insulin sensitivity^6,7,32^, making exercise an efficacious treatment for type 2 diabetes mellitus and numerous other chronic conditions^2^. However, at the population level, the adherence to exercise training is poor. As we are facing an unprecedented obesity crisis with serious comorbidities, like diabetes, deciphering the molecular blueprint that underpins the metabolic benefits of exercise possesses therapeutic potential.

Therefore, we characterized global protein changes in response to ET. The inclusion of HFD and ET on HFD groups is a major strength of this study. Both ET and HFD increase lipid oxidative capacity in skeletal muscle^20,21^, but only ET leads to health benefits, suggesting that proteins similarly regulated by ET and HFD are unlikely candidates to transduce the beneficial effects of exercise training. Thus, we were interested in identifying proteins that are differentially regulated by HFD and ET, such as MUP1, which was markedly decreased by HFD but increased with ET.

MUP1 belongs to the lipocalin family, which comprises a large group of small extracellular proteins with diverse functions. Lipocalins have been shown to bind and transport small hydrophobic molecules as well as soluble macromolecules and some lipocalins bind to specific cell-surface receptors^33^. For example, lipocalin 2, secreted from bone, is thought to act on the melanocortin 4 receptor (MC4R) in the hypothalamus to reduce appetite^34^. MUP1 is thought to be predominantly expressed in and secreted from the liver and circulating MUP1 levels are decreased in mouse models of obesity^26,27^. Pharmacologically restoring MUP1 levels in male obese db/db mice improves whole body insulin sensitivity^27^. How this is mediated by MUP1 and on what organs MUP1 is acting is unknown. Our data indicate that MUP1 is expressed in skeletal muscle, and, akin to the circulating MUP1 levels, protein abundance is down-regulated in obesity. Interestingly, we show that MUP1 improves insulin sensitivity of GLUT4 translocation in cultured muscle cells, but it is unclear if this is mediated via specific receptors or if MUP1 is taken up by the muscle to trigger signaling events inside the cell. Our signaling data appear to rule out MUP-1 effects on Akt, AMPK and p38, which all have been implicated in the regulation of GLUT4 translocation in muscle^35^. Alternatively, MUP1 could also act as a binding partner that is required for other proteins to function. We also found that the ET-dependent induction of *Mup1 gene* expression required functional AMPK. Since AMPK is not necessary for Mup1 expression in the sedentary state, it is most likely that AMPK-specific pathways activated with exercise regulate *Mup1* expression, but the details of this regulation remain to be unraveled.

In addition to MUP1, two other hits stand out to us. One is the SRA Stem-Loop Interacting RNA Binding Protein (SLIRP), which has recently been identified as a novel regulator of mitochondrial protein translation^36^. In our study, SLIRP was increased with both ET and ET on HFD, while HFD alone had no effect on SLIRP protein abundance. This leads to the intriguing hypothesis that mitochondrial adaptations and possibly improvements in exercise performance with ET are in part regulated by SLIRP. Another intriguing hit is the guanylate binding protein 4 (GBP4), which was increased with HFD whereas both ET and ET during HFD decreased GBP4 protein. GBPs are thought to play a role in the innate immune response and their expression is generally increased by pro-inflammatory stimuli^37^. Thus the expression pattern may reflect an HFD-mediated inflammatory state that can be ameliorated by ET. Whether genetic downregulation of the protein would confer metabolic protection during HFD feeding in skeletal muscle would be an interesting hypothesis to test.

A limitation of our findings is the use of an animal model. Although there is considerable overlap between humans and mice in metabolic processes and regulation thereof, there are also considerable distinctions, such as the vast difference in metabolic rate between mice and humans^17^. Therefore, it remains to be seen whether exercise and diet regulate the same proteins and whether these proteins in turn regulate distinct metabolic processes in human skeletal muscle. This, however, can now be addressed following our contribution in targeted hypothesis driven follow-up studies.

In conclusion, since obesogenic HFD and exercise training have opposite impact on health, we believe that proteins that are differentially regulated by exercise training and HFD are particularly promising candidate proteins to transduce exercise-induced health benefits. This rationale is supported by the identification of MUP1 as a novel diet-, ET- and AMPK-regulated skeletal muscle protein that improves insulin sensitivity of GLUT4 translocation in muscle cell culture. This warrants future investigations into MUP1 and also into the other unique molecular targets we have provided.

## Methods

### Animals

Female C57BL/6J (Taconic) mice with or without access to running wheels received standard chow diet (Altromin No. 1324; Brogaarden, Denmark), or 60% high-fat diet (HFD) (D12492; Brogaarden, Denmark) and water ad libitum. Mice, 9 weeks old at the start of the 20 weeks intervention, were single-housed on a 12:12-h light-dark cycle at 21–22 degrees Celsius. The Danish Animal Experimental Inspectorate approved all experiments and all experiments were performed in accordance with relevant guidelines and regulations. For the AMPK KD and wild-type mice, information on animal care and experimental design can be found here^30^.

### *In vivo* 2-deoxy-glucose (2DG) uptake experiments

Mice were fasted for 3–5 hour from 7 AM and anaesthetized with pentobarbital (i.p. injection of 7.5/9.5 mg (for chow/HFD-fed mice) pentobarbital sodium per 100 g body weight). A bolus of saline containing 66.7 μCi/ml [^3^H]2DG (Perkin Elmer) corresponding to ∼9 μCi/mouse (6 μl/g body weight) was injected retro-orbitally. In addition, the injectate contained either 0.5U/kg body weight insulin (Actrapid, Novo Nordisk, Bagsværd, Denmark) or a comparable volume of saline as in^38^. Blood was collected from the tail vein before and 5 and 10 min after the injection. At the same time, a drop of blood was used to measure glucose concentration with a handheld glucometer (Bayer Contour, Switzerland). Specific 2-[^3^H]DG tracer activity was analyzed in the 10 min blood sample. After 10 min mice were killed by cervical dislocation and gastrocnemius muscle were excised, snap-frozen in liquid nitrogen and stored at −80 °C. 2DG uptake was analyzed as described before^39^.

### Body composition analysis

Fat mass and lean body mass were determined with an Echo MRI scanner 18 weeks into the 20-week intervention.

### Proteomic analysis

Quantitative proteomics was performed on a total of 18 mouse gastrocnemius muscle samples from; (i) chow sedentary (n = 4), (ii) chow training (n = 4), (iii) HFD sedentary (n = 5), and iv) HFD training (n = 5). Muscles were lysed in 6 M urea, 2 M thiourea, 0.1% SDS in 50 mM triethylammonium bicarbonate (TEAB) pH 8.0 containing a protease and phosphatase inhibitor cocktail (Roche) using tip-probe sonication. The lysates were centrifuged at 16,000 × g for 10 min and protein precipitated with 5 volumes of acetone overnight at −30 °C. The precipitated proteins were resuspended in 6 M urea, 2 M thiourea in 50 mM TEAB pH 8.0 and 100 µg of protein normalised to a final volume of 40 µL. The proteins were reduced with 10 mM dithiothreitol (DTT) for 60 min at room temperature followed by 25 mM iodoacetamide for 30 min at room temperature in the dark. The reactions were quenched to a final concentration of 20 mM DTT and digested with 2 µg of Lys-C at room temperature for 2 h. The digest was diluted with 5 volumes of 50 mM TEAB pH 8.0 and digested with 2 µg of trypsin overnight at 37 °C. The digest was acidified to a final concentration of 2% formic acid and centrifuged at 16,000 × g for 10 min. Peptides were desalted with hydrophilic-lipophilic solid phase extraction (HLB-SPE; Waters) followed by elution with 50% acetonitrile containing 0.1% trifluoroacetic acid (TFA) and dried by vacuum centrifugation. Peptides were resuspended in 25 µL of 500 mM TEAB pH 8.3 and normalised to 10 µg in 8 µL. Peptides were labelled by adding 0.1 mg TMT reagent in 8 µL of acetonitrile and incubated at room temperature for 90 min. The reactions were quenched with 1 µL of 5% hydroxylamine and 150 µL of 0.1% TFA followed by pooling of the peptides to result in two multiplexed experiments which were desalted with HLB-SPE. Each experiment was fractionated into 12 fractions using hydrophilic interaction liquid chromatography as previously described^40^.

Each fraction was analysed on a Dionex 3500RS nanoUHPLC coupled to an Orbitrap Fusion mass spectrometer with Tune v1.2.1149 in positive mode. Peptides were separated using an in-house packed 75 μm × 40 cm pulled column (1.9 μm particle size, C18AQ; Dr Maisch, Germany) with a gradient of 2–30% MeCN containing 0.1% FA over 120 min at 250 nl/min at 55 °C. An MS1 scan was acquired from 375–1575 m/z (120,000 resolution, 4e5 AGC, 100 ms injection time) followed by MS2 data-dependent acquisition of the 12 most intense precursor ions with CID and detection in the ion trap (2e4 AGC, 70 ms injection time, 35 NCE, 2.0 m/z quadrupole isolation width, 0.25 activation Q). To quantify TMT reporter ions, a synchronous precursor selection MS3 scan was performed on the 10 most abundant fragment ions with HCD and detection in the orbitrap (100–500 m/z, 1e5 AGC, 120 ms injection time, 55 NCE, 2.0 m/z isolation width)^41^.

### Analysis of mass spectrometry data

All raw data were processed with Proteome Discoverer v2.1.0.81 and searched against the mouse UniProt database using SequestHT. The MS1 tolerance was set to 20 ppm and the MS2 mass tolerance set to 0.6 Da. Peptides were searched with a maximum of 2 miss cleavages with oxidation of methionine set as a variable modification and, carbamidomethylation of cysteine and TMT labelling of peptide N-terminus/lysine set as fixed modifications. All data was filtered to a 1% peptide spectral match (PSM) FDR and protein FDR using percolator. Protein identification and quantification was performed with a minimum of one peptide. Reporter ion quantification was performed using 20 ppm and, PSMs filtered for a signal-to-noise greater than 10 and a maximum co-isolation threshold of 50%. Statistical analysis was performed in Perseus software and differentially regulated proteins calculated using t-tests corrected for multiple testing with Benjamini Hochberg adjustment. Significantly regulated proteins were defined with an adjusted p, q < 0.05 and fold-change +/−50%.

### Gene ontology (GO) term enrichment analysis

Gene names of significantly regulated proteins (q < 0.05 and fold-change of +/−50%) were submitted to a PANTHER Overrepresentation Test online (http://www.pantherdb.org) using the PANTHER version 13.0 (released 2017-11-12) with a FISHER test type and a *Mus musculus* reference list. Over-presented PANTHER GO-Slim Biological Process terms with a false discovery rate of less than one percent on a log10 enrichment of >0.5 are shown as relative font sizes.

### Preparation of RNA and gene expression analysis

Total RNA from tissues was prepared using RNeasy Kit (Qiagen, Hilden, Germany) according to the manufacturer’s instructions. cDNA synthesis was performed with a QuantiTect Reverse Transcription Kit (Qiagen, Hilden, Germany) according to the manufacturer’s instructions. Gene expression was profiled with SYBR Green based quantitative PCR (qPCR), using the 7900HT Fast Real-Time PCR System (Thermo Fisher Scientific, Erlangen, Germany). The relative expression levels of each gene were normalized to the housekeeping gene hypoxanthine phosphoribosyltransferase (Hprt) using the ddCt method. The following primers were used: Mup1 forward 5′-GAAGCTAGTTCTACGGGAAGGA-3′, Mup1 reverse 5′-AGGCCAGGATAATAGTATGCCA-3′ and Hprt forward: 5′-AAGCTTGCTGGTGAAAAGGA-3′, Hprt reverse 5′-TTGCGCTCATCTTAGGCTTT-3′. Primer efficiency was tested.

### MUP1 treatment and GLUT4 translocation in L6 cells

L6 myoblasts stably over-expressing GLUT4 with a c-myc epitope tag (L6-GLUT4myc)^42^, a kind gift from Amira Klip, were grown in α-MEM media (GIBCO #1257-063) with 10% fetal bovine serum (GIBCO #26140-038), 100 μg/mL streptomycin, 100 units/mL penicillin, 0.25 μg/ml Fungizone^©^ (GIBCO #15240-062) (5% CO_2_, 37 °C). Cells were seeded in 96 well plates and grown until 90% confluent. Cells were incubated with the indicated concentrations of recombinant MUP1 protein (Abcam; Cat# ab95193) for 24 hours. For analysis of GLUT4 translocation, cells were serum starved for 4 hours and stimulated with insulin (10 minutes, submaximal dose of 10 nM) or not stimulated. Immediately following stimulations, cells were chilled on ice, fixed with 3% paraformaldehyde, blocked in 5% goat serum, and incubated with primary anti-myc antibody (Cell Signaling Technology). The signal was detected with a secondary HRP-antibody and o-Phenylenediamine reagent (Sigma–Aldrich) was added to each well to initiate a color reaction with the secondary antibody. This was terminated with 5 M HCl, before absorbance at 492 nm was measured. Background absorbance (no primary antibody) was subtracted. The experiment was performed twice.

For the signaling experiment, cells were treated exactly as above, except that cells were seeded in 12-well plates and at the end of insulin stimulation cells were lysed for western blotting. The signaling experiment was performed three times and a representative experiment is shown in the manuscript.

### Western Blotting

Muscles were pulverized in liquid nitrogen and homogenized 2 × 0.5 min at 30 Hz using a Tissuelyser II bead mill (Qiagen, USA) in ice-cold homogenization buffer (10% Glycerol, 20 mM Na-pyrophosphate, 150 mM NaCl, 50 mM HEPES (pH 7.5), 1% NP-40, 20 mM β-glycerophosphate, 10 mM NaF, 2 mM PMSF, 1 mM EDTA (pH 8.0), 1 mM, EGTA (pH 8.0), 10 μg/ml Aprotinin, 10 μg/ml Leupeptin, 2 mM Na3VO4, 3 mM Benzamidine, 5 mM Nicotinamide). Homogenates were rotated end-over-end for 1 h at 4 °C, and cleared by centrifugation at 10000 × g for 20 min at 4 °C. Cells were washed once with ice-cold PBS, quickly dried, and scraped off in presence of 150 μl ice-cold RIPA Buffer (SIGMA, Cat# R0278) with protease and phosphatase inhibitor (ThermoFisher, Cat# 78446). Cells were rigorously pipetted up and down 10 times and then centrifuged at 13000 × g for 10 min at 4 °C. Muscle and cell lysate protein content was determined by bicinchoninic acid method and lysates were diluted to the same protein concentration. Total protein and phosphorylation levels of indicated proteins were determined by standard immunoblotting, loading equal amounts of protein. The following antibodies were used:

**Table:** 

Protein	Company	Cat. #
MUP1	Santa Cruz Biotechnology	SC-166429
SLIRP	Novus Biologicals	NB110-37258
Myoglobin	Cell Signaling Technology	25919
ACOT13	Cell Signaling Technology	42713
pAKT 308	Cell Signaling Technology	9275
pAKT473	Cell Signaling Technology	4058
pTBC1D4 642	Cell Signaling Technology	8881
pACC 212	Cell Signaling Technology	3661
pP38 MAPK 180/182	Cell Signaling Technology	9211
Actin	Cell Signaling Technology	4967

### Statistical analysis of non-proteome data

Results are shown means ± SEM. Statistical testing was performed using paired t-tests or one- or two-way ANOVA (repeated or non repeated measurements) as appropriate. Tukey’s post hoc test was performed when ANOVA revealed significant interaction. The significance level was set at p < 0.05.

## Electronic supplementary material

Raw blots related to figure 4

Raw blots related to figure 5

Supplemental Figure 1

Supplemental Dataset 1
