# Supplementary material for: Quantitative proteomic characterization of cellular pathways associated with altered insulin sensitivity in skeletal muscle following high-fat diet feeding and exercise training
Source: Sci Rep. 2018 Jul 16;8:10723. doi: 10.1038/s41598-018-28540-5 (PMC6048112; doi:10.1038/s41598-018-28540-5)

Quantitative proteomic characterization of cellular pathways associated with altered insulin sensitivity in skeletal muscle following high-fat diet feeding and exercise training

Maximilian Kleinert[1,2,3,4], Benjamin L. Parker[5], Thomas E. Jensen[1], Steffen H. Raun, Phung Pham[2], Xiuqing Han[1], David E. James[5], Erik A. Richter[1], Lykke Sylow[1#]

Supplemental Info – Raw Blots related to Figure 4

ACOT13

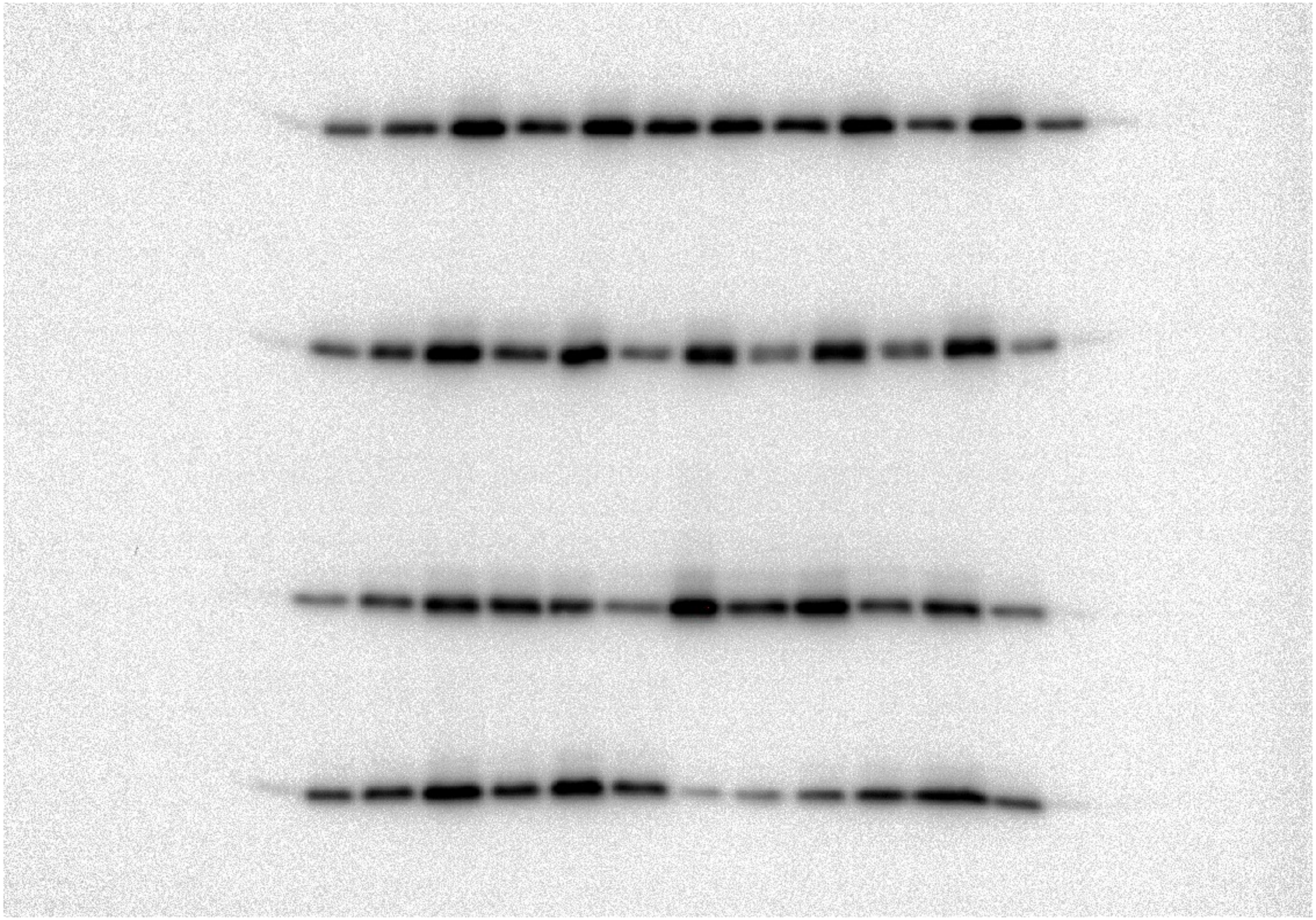

SLIRP

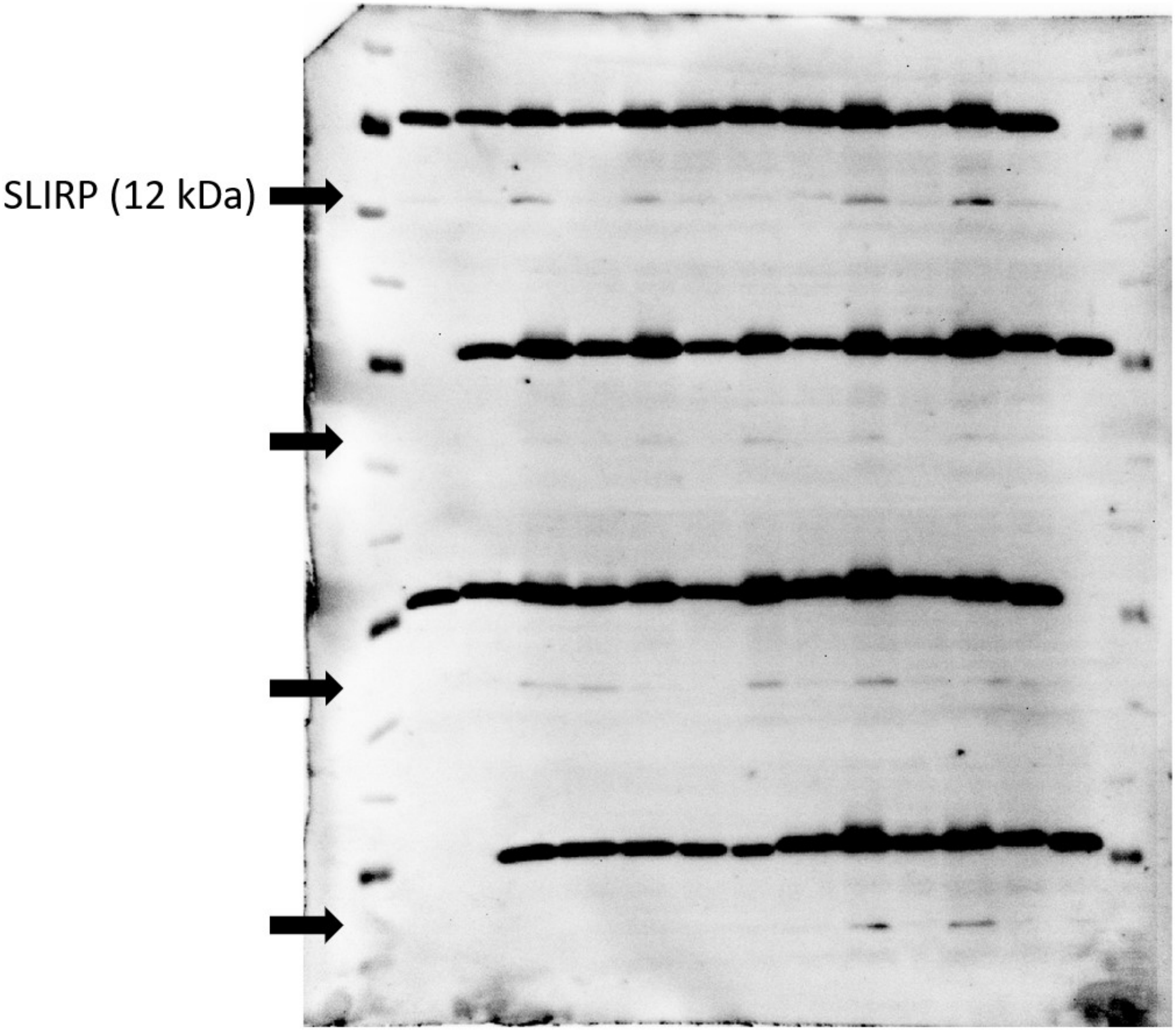

MUP1

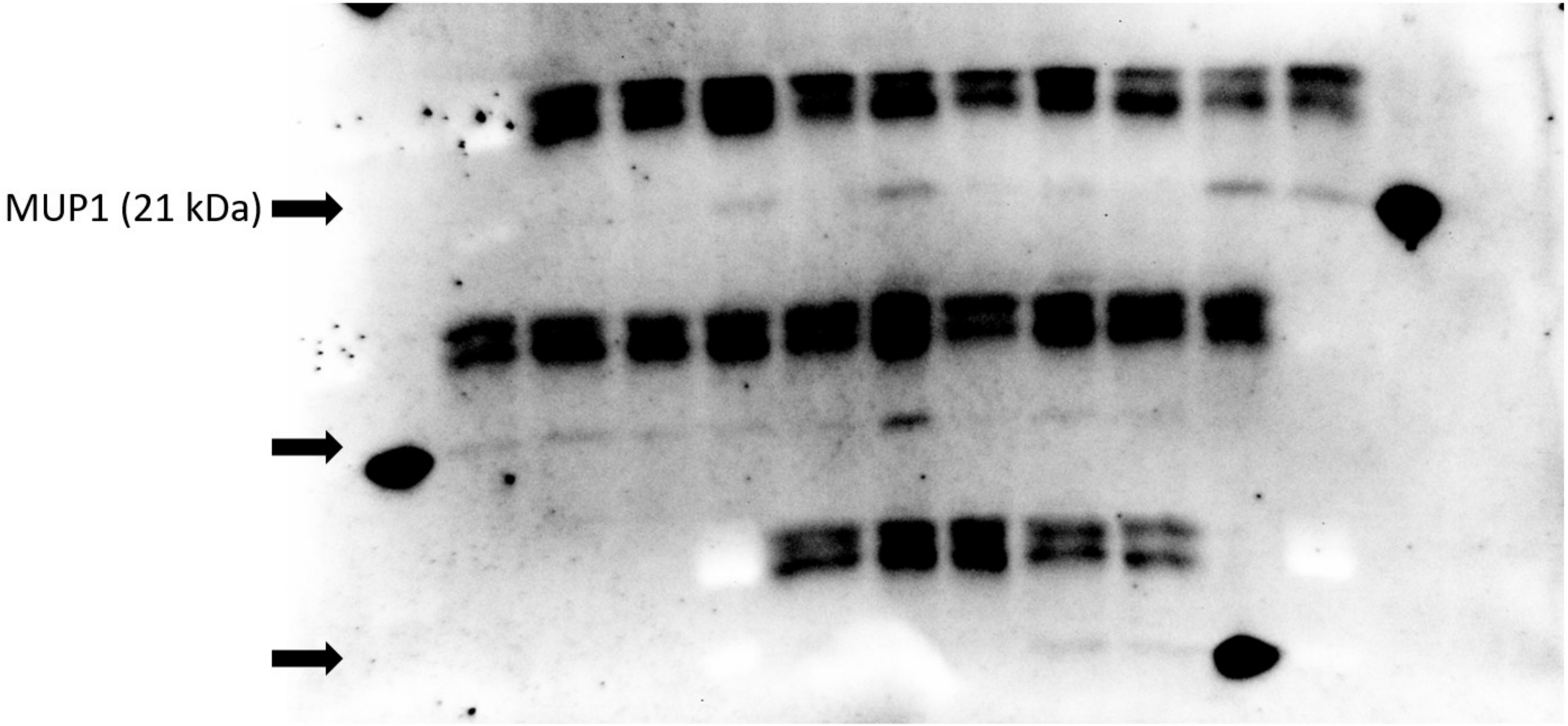

Myoglobin

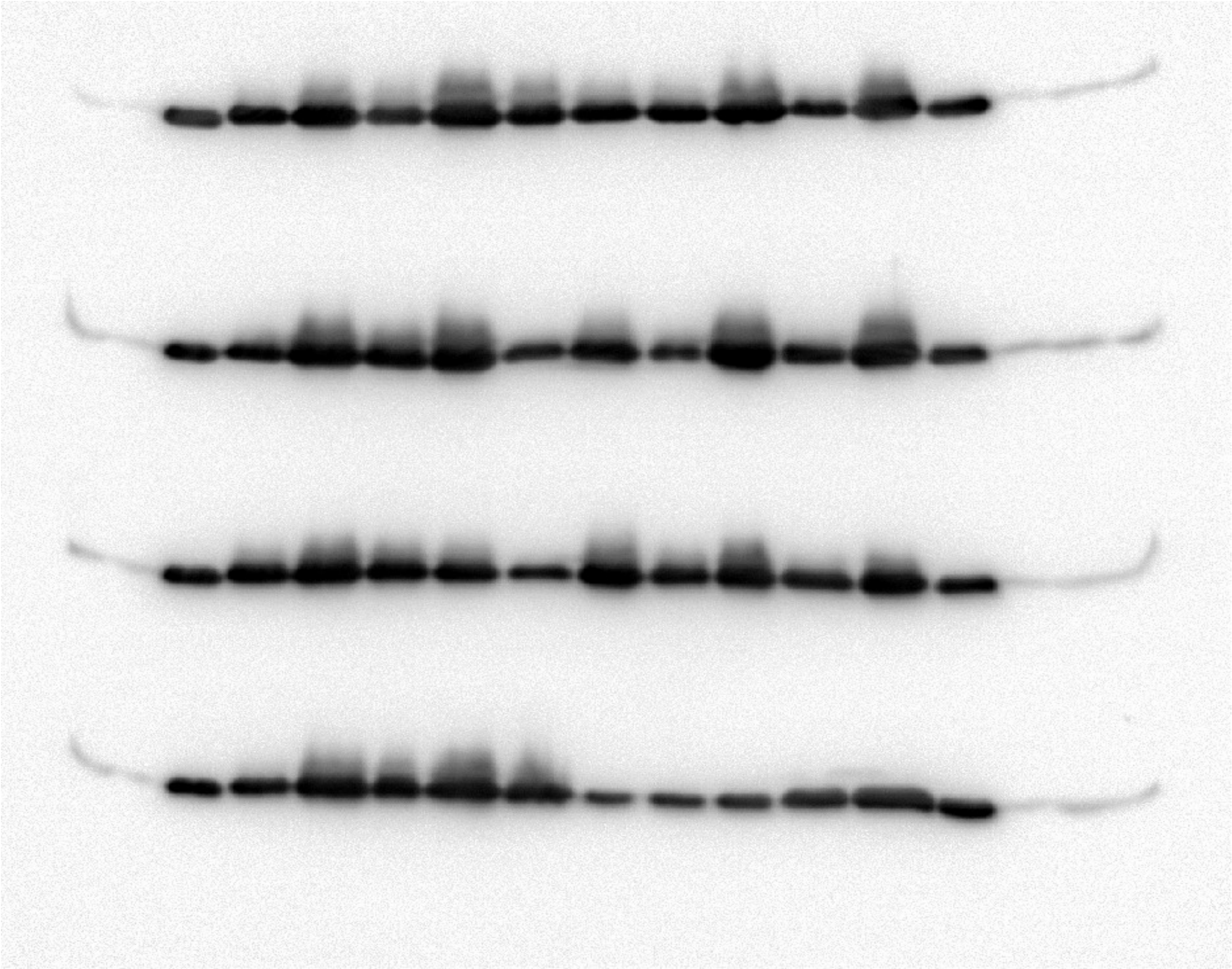

Coomassie Stain

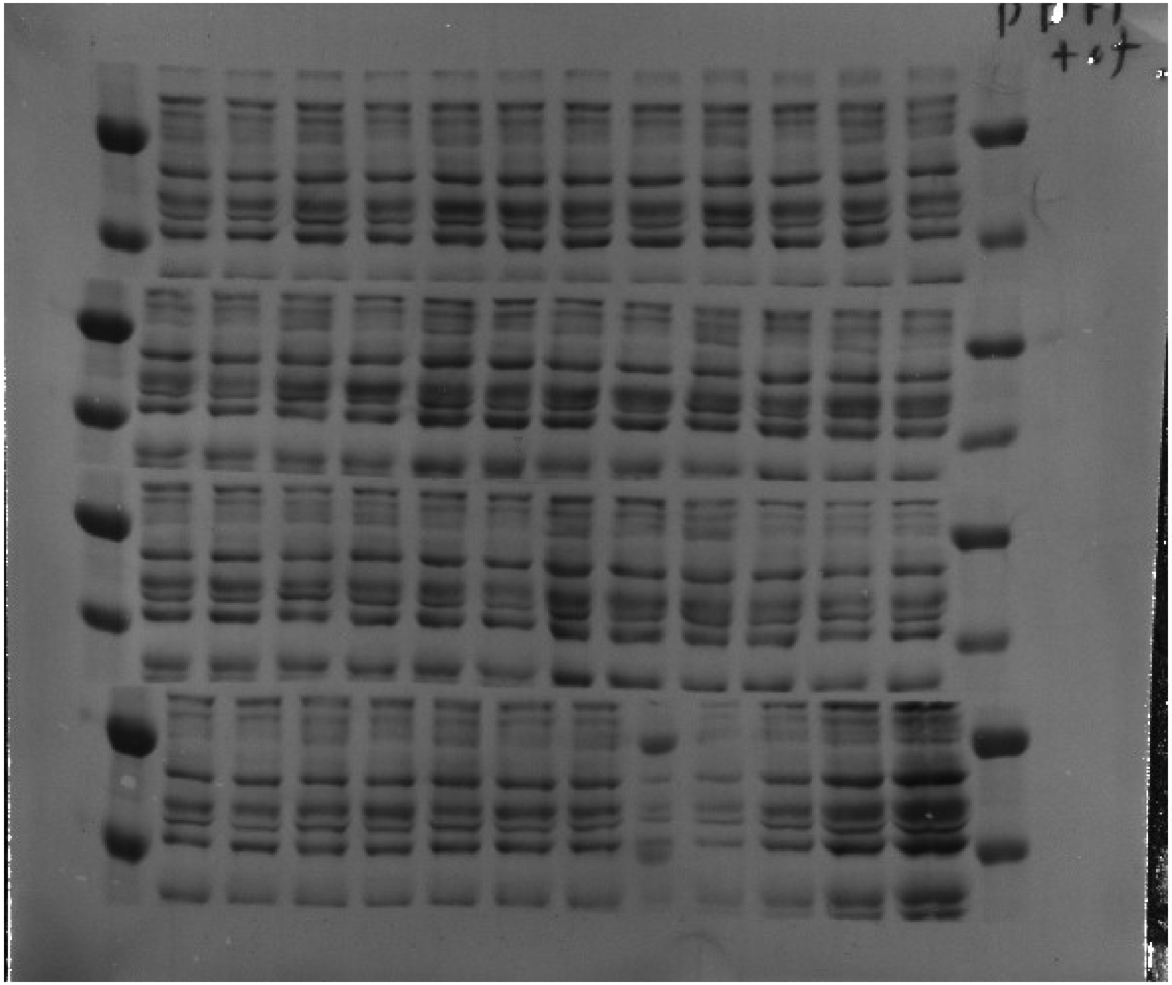

Supplement: Supplementary file 1 — Raw blots related to figure 4 [file 41598_2018_28540_MOESM1_ESM.pdf]
