# Supplementary material for: Quantitative proteomic characterization of cellular pathways associated with altered insulin sensitivity in skeletal muscle following high-fat diet feeding and exercise training
Source: Sci Rep. 2018 Jul 16;8:10723. doi: 10.1038/s41598-018-28540-5 (PMC6048112; doi:10.1038/s41598-018-28540-5)

Quantitative proteomic characterization of cellular pathways associated with altered insulin sensitivity in skeletal muscle following high-fat diet feeding and exercise training

Maximilian Kleinert[1,2,3,4], Benjamin L. Parker[5], Thomas E. Jensen[1], Steffen H. Raun, Phung Pham[2], Xiuqing Han[1], David E. James[5], Erik A. Richter[1], Lykke Sylow[1#]

Supplemental Info – Raw Blots related to Figure 5

p-ACC

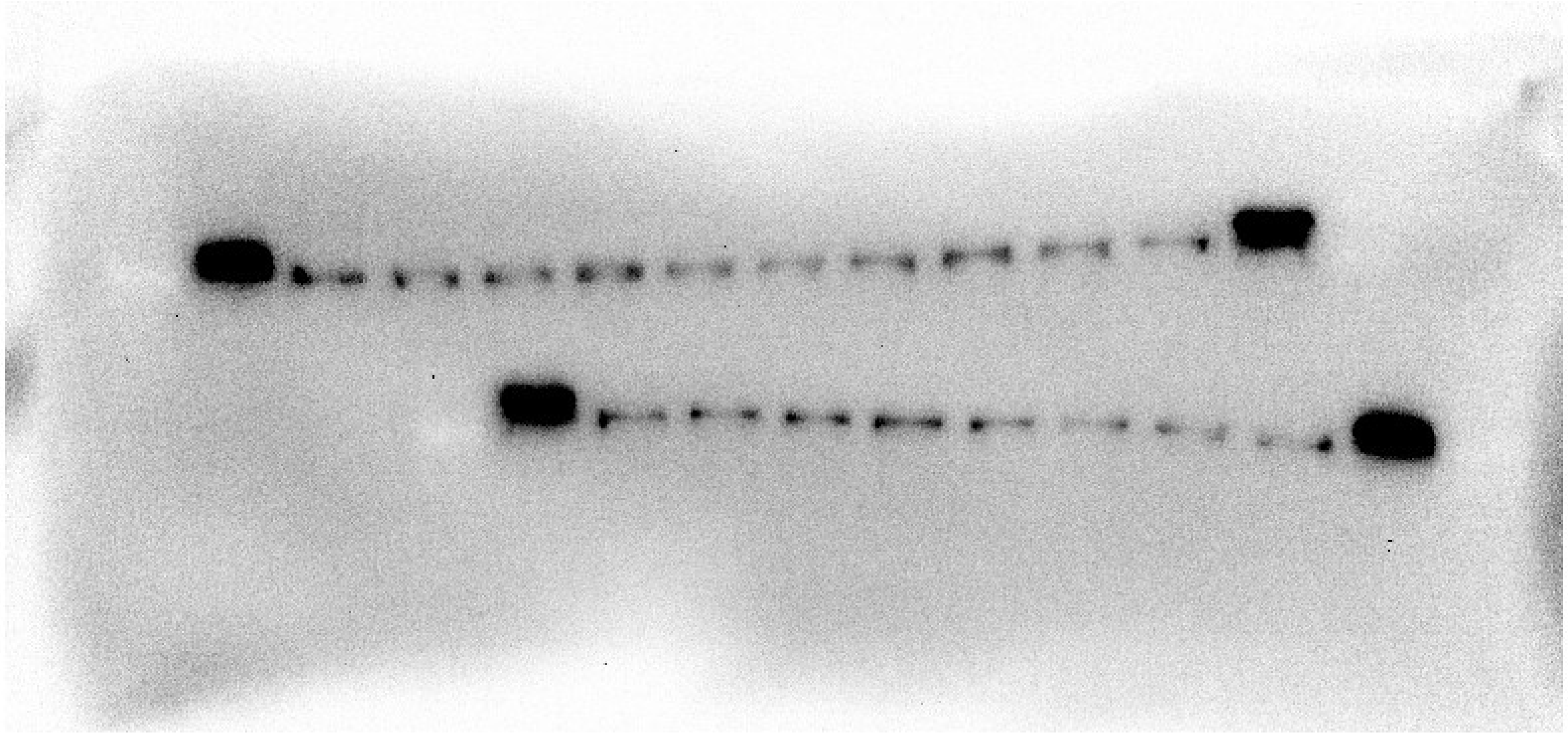

Actin

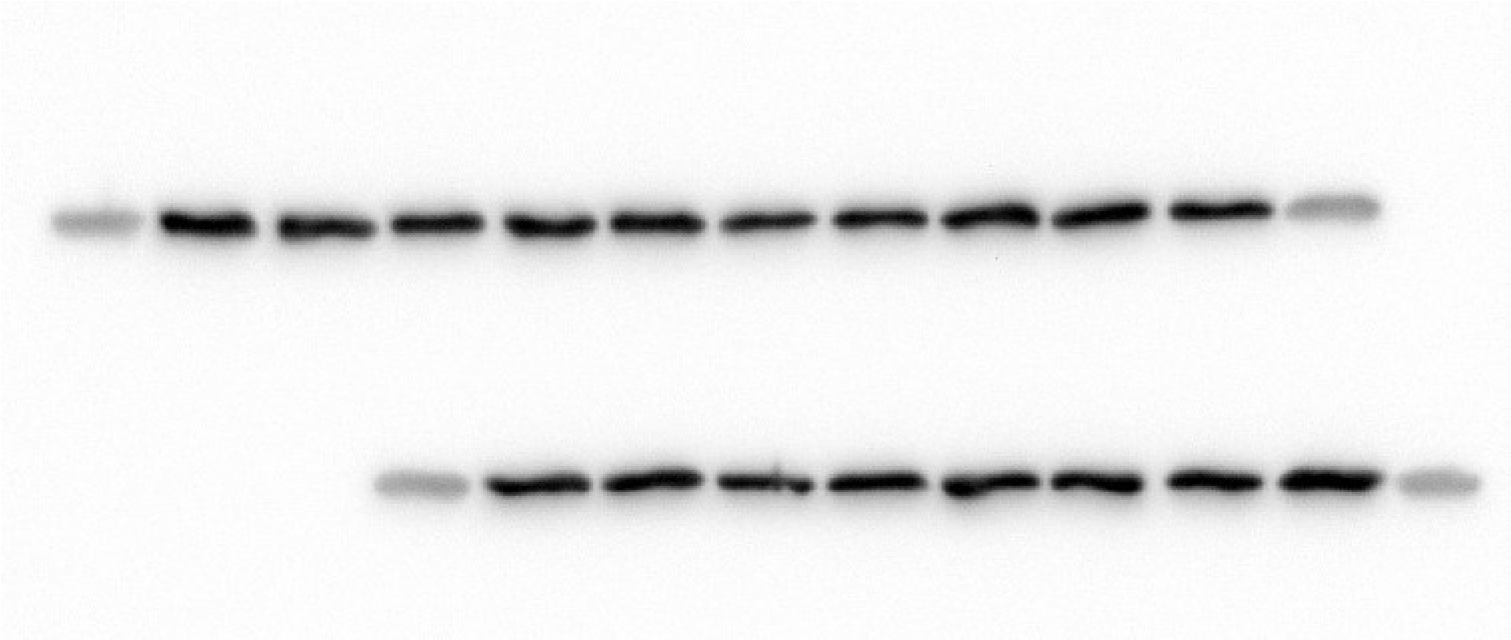

p-AKT T308

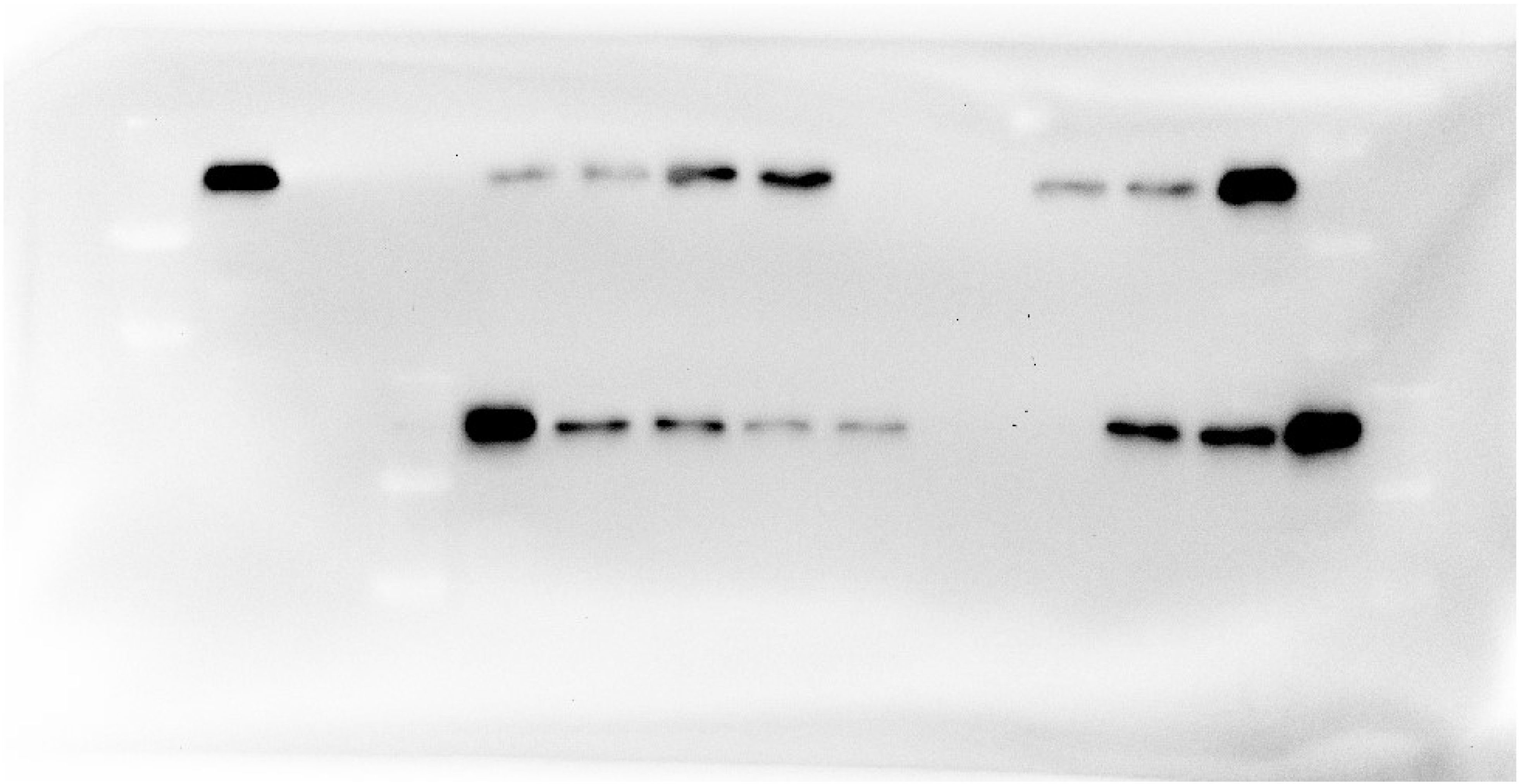

p-Akt S473

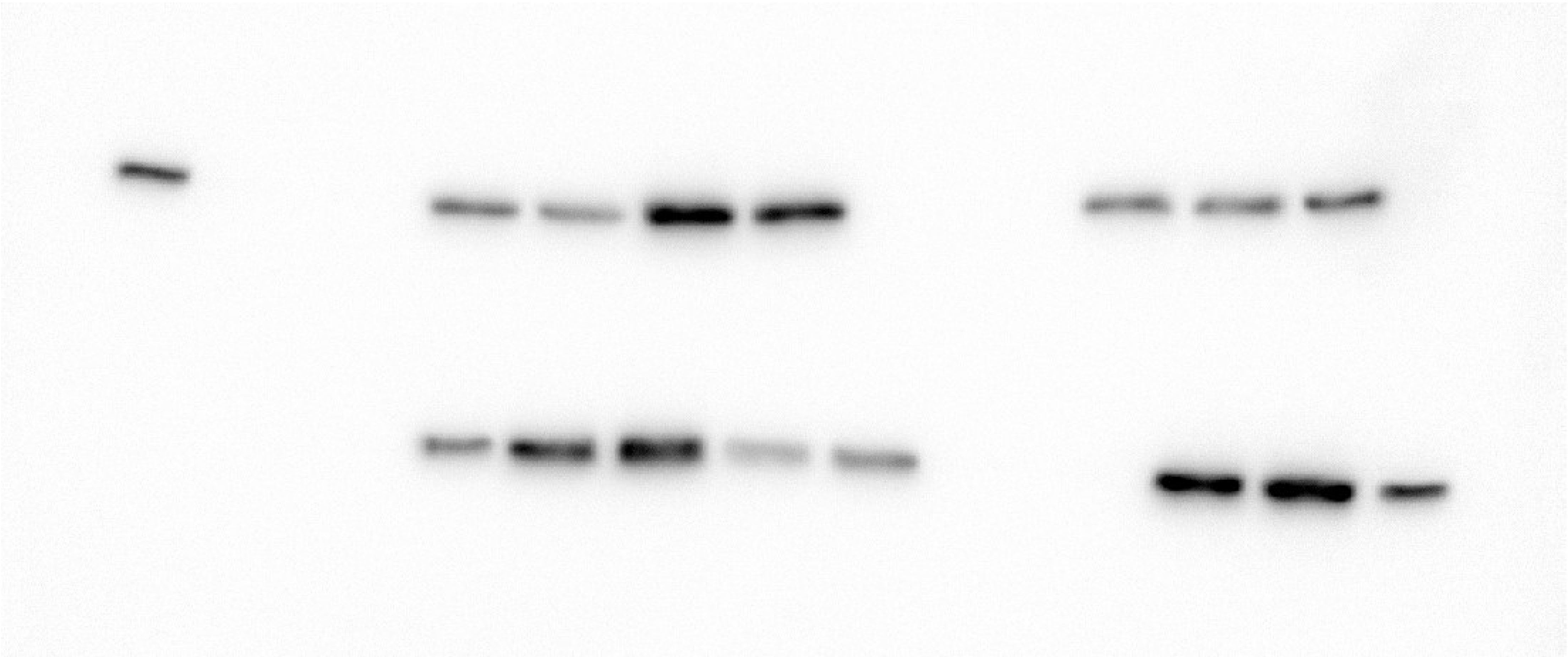

p-TBC1D4 T642

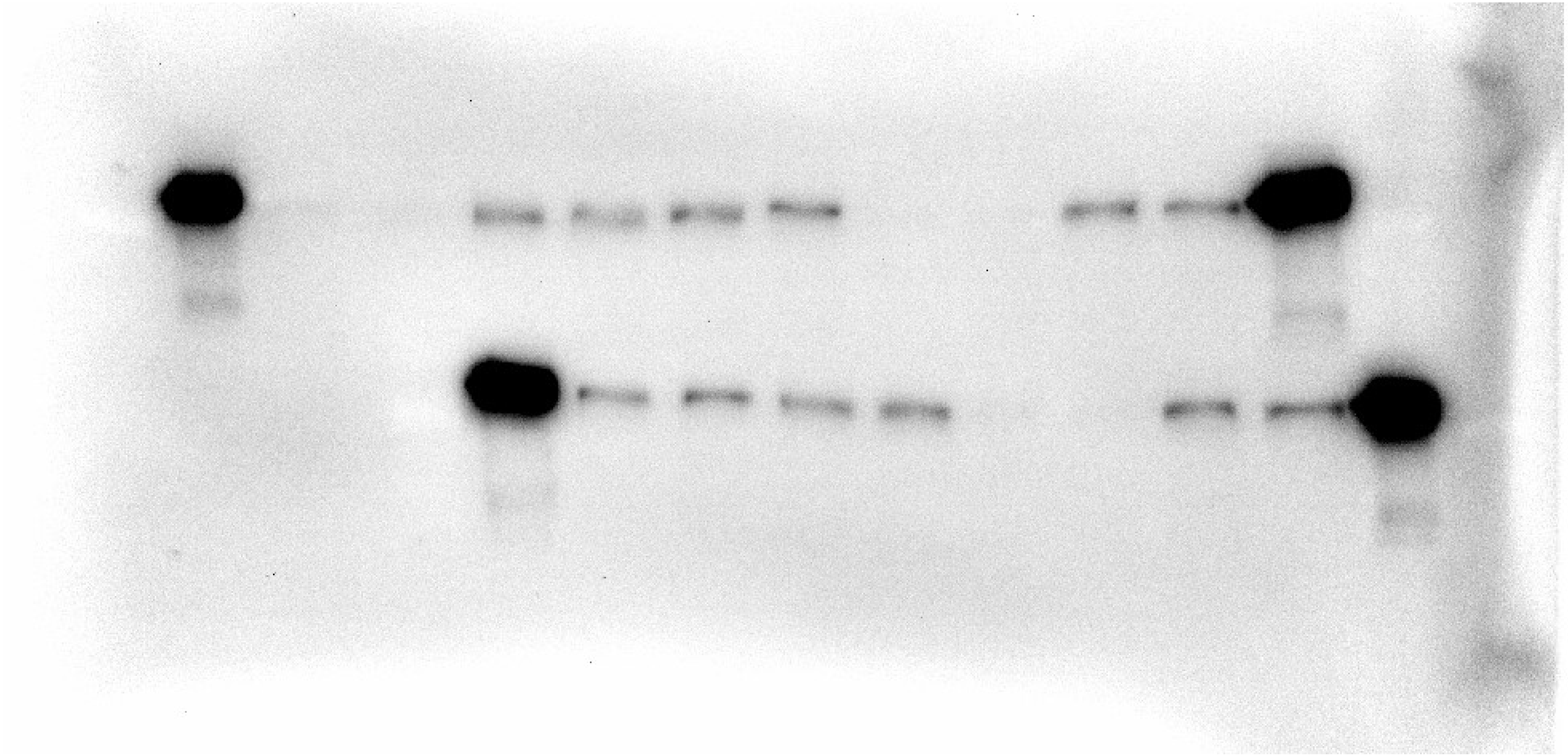

p-P38

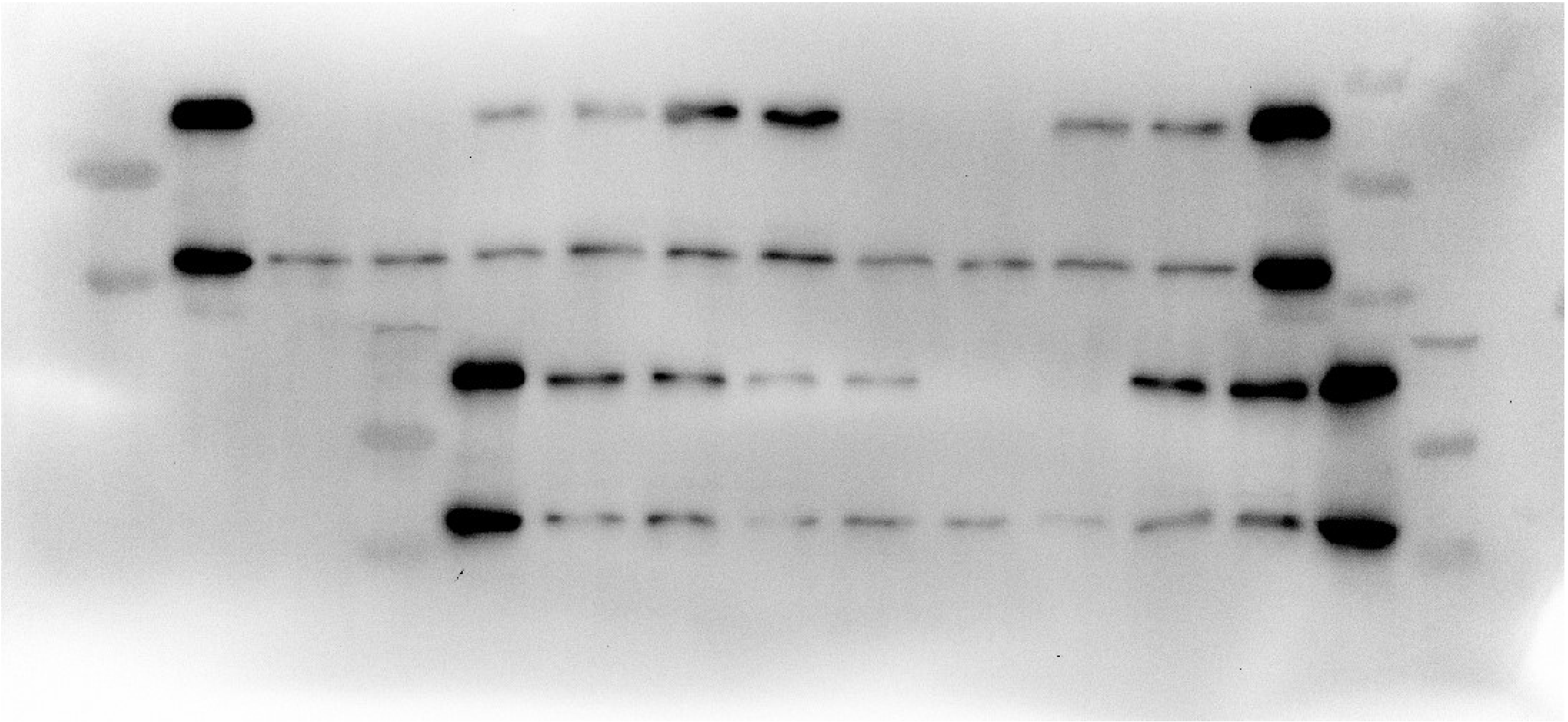

Supplement: Supplementary file 2 — Raw blots related to figure 5 [file 41598_2018_28540_MOESM2_ESM.pdf]
