# Supplementary material for: Quantitative proteomic characterization of cellular pathways associated with altered insulin sensitivity in skeletal muscle following high-fat diet feeding and exercise training
Source: Sci Rep. 2018 Jul 16;8:10723. doi: 10.1038/s41598-018-28540-5 (PMC6048112; doi:10.1038/s41598-018-28540-5)
Supplement: Supplementary file 3 — Supplemental Figure 1 [file 41598_2018_28540_MOESM3_ESM.pdf]

Supplemental Figure

Quantitative proteomic characterization of cellular pathways associated with altered insulin sensitivity in skeletal muscle following high-fat diet feeding and exercise training

Maximilian Kleinert[1,2,3,4], Benjamin L. Parker[5], Thomas E. Jensen[1], Steffen H. Raun, Phung Pham[2], Xiuqing Han[1], David E. James[5], Erik A. Richter[1], Lykke Sylow[1#]

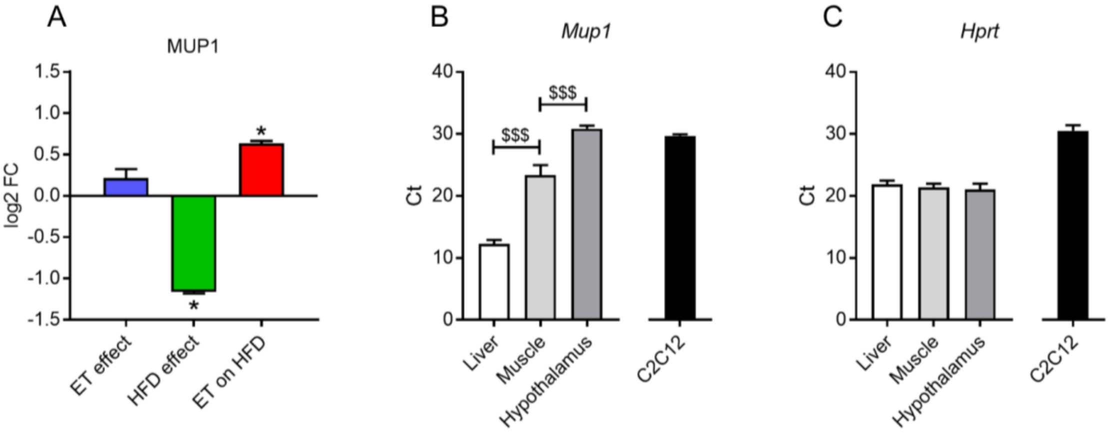

Supplemental Figure 1. MUP1 protein regulation, as detected in the proteome, by the indicated interventions (A). Cycle threshold (Ct) values for *Mup1* and *Hprt* (used as a housekeeper) (B,C) in indicated tissues (n = 3-5). Data are means +/- SEM. $^{\$\$\$}p < 0.001$ between the indicated groups. *q < 0.05 and fold-change +/-50%.
